# Pathogenic free-living amoebae from water sources in Cape Verde

**DOI:** 10.1007/s00436-022-07563-y

**Published:** 2022-06-04

**Authors:** Djeniffer Sousa-Ramos, María Reyes-Batlle, Natalia Karla Bellini, Rubén L. Rodríguez-Expósito, Christian Martín-Real, José E. Piñero, Jacob Lorenzo-Morales

**Affiliations:** 1 Instituto Universitario de Enfermedades Tropicales Y Salud Pública de Canarias (IUETSPC), Universidad de La Laguna (ULL), Avda. Astrofísico Fco. Sánchez S/N, 38203 La Laguna, Tenerife Spain; 2 Departamento de Obstetricia Y Ginecología, Pediatría, Medicina Preventiva Y Salud Pública, Toxicología, Medicina Legal Y Forense Y Parasitología, Universidad de La Laguna (ULL), San Cristóbal de La Laguna, Tenerife, Spain; 3 Red de Investigación Cooperativa en Enfermedades Tropicales (RICET), Madrid, Spain; 4 Instituto de Física de São Carlos, Universidade de São Paulo, Caixa Postal 369, São Carlos, SP 13560-590 Brazil; 5 Consorcio Centro de Investigación Biomédica En Red M.P. (CIBER) de Enfermedades Infecciosas (CIBERINFEC), Inst. de Salud Carlos III, 28006 Madrid, Spain

**Keywords:** Cape Verde, Water, *Acanthamoeba* spp., *Vermamoeba vermiformis*, *Vannella* spp.. *Stenamoeba dejonckheerei*

## Abstract

Free-living amoebae (FLA) are protozoa which have been reported in different countries worldwide from diverse sources (water, soil, dust, air), contributing to the environmental microbiological contamination. Most of the FLA species present a life cycle with two different phases: an active vegetative and physiologically form named trophozoite, and an extremely resistant phase called cyst. *Acanthamoeba* spp., *Naegleria fowleri*, *Balamuthia mandrillaris*, *Sapinia pedata*, *Vahlkampfia* spp., *Paravahlkampfia* spp. and *Vermamoeba vermiformis* have been reported not only as causal agents of several opportunistic diseases including fatal encephalitis or epithelial disorders, but also as capable to favour the intracellular survival of common pathogenic bacteria, which could avoid the typical water disinfection systems, non-effective against FLAs cysts. Even though Santiago Island possesses high levels of humidity compared to the rest of the archipelago of Cape Verde, the water resources are scarce. Therefore, it is important to carry out proper microbiological quality controls, which currently do not contemplate the FLA presence in most of the countries. In the present work, we have reported the presence of *Acanthamoeba* spp. (69.2%); *Vannella* spp. (15.4%); *Vermamoeba vermiformis* (7.7%) and the recently discovered *Stenamoeba dejonckheerei* (7.7%) in different water sources of Santiago Island.

## Introduction

Cabo Verde Archipelago is located on the west coast of Africa, and consists in 10 islands divided in two groups: the Barlavento group (Santo Antão, São Vicente, Santa Luzia, São Nicolau, Sal and Boavista islands) and the Sotavento group (Maio, Santiago, Fogo and Brava islands). Nevertheless, each island presents topographic and climatic differences, promoting historically the active movement of population and goods (Leal et al. 2020). Cape Verde’s climate is milder than the African mainland, due to the surrounding sea which moderates temperatures of the islands and the cold Atlantic currents which produce an arid atmosphere around the archipelago. However, the islands do not receive the upwellings (cold streams) that affect the West African coast, so the air temperature is cooler than in Senegal. Nevertheless, the sea is warmer, because the orographic relief of some islands, such as Santiago with steep mountains, covers it with rich woods and luxuriant vegetation where the humid air condenses and soaks the environment (Coverdell 2009). Due to this water availability, one of the main industries in the island is agriculture, followed by tourism, fishing and others, alongside some manufacturing. However, even though Santiago Island possesses high levels of humidity compared to the rest of the archipelago, the water resources are scarce. Consequently, it is important to highlight the importance of proper microbiological quality controls.

The widely reported protozoa free-living amoebae (FLA) have been reported in different water sources, soils or air among others (Schuster and Visvesvara 2004). Moreover, their contribution to the environmental microbiological contamination has been demonstrated (Guimaraes et al. 2016). *Acanthamoeba* spp., *Naegleria fowleri*, *Balamuthia mandrillaris*, *Sapinia pedata*, *Vahlkampfia* spp., *Paravahlkampfia* spp. and *Vermamoeba vermiformis* have been reported as causal agents of several opportunistic diseases including fatal encephalitis or epithelial disorders (Scheid et al. 2019; Schuster and Visvesvara 2004; Siddiqui et al. 2021). The life cycle of these pathogenic microorganisms is formed by a physiologically active vegetative trophozoite and an extremely resistant and persistent stage called cyst (Siddiqui and Khan 2012). Furthermore, this resistant stage can favour the intracellular survival of common pathogenic bacteria, avoiding the typical water disinfection systems, non-effective against FLAs cysts (Lorenzo-Morales et al. 2015).

Regarding the quality of water on the island of Santiago, there is a regulatory decree number 5/2017 of 6 November that establishes the criteria and standards that define the essential requirements for the quality of water intended for human consumption, as well as the control systems, the sanctioning regime and protective measures, with a view to protecting human health from the adverse effects resulting from possible water contamination. However, for irrigation systems where the water comes from springs and boreholes used in this study, there is still no quality control to evaluate the physical, chemical and microbiological parameters weekly or quarterly. Therefore, the aim of our study was to evaluate the presence of FLA from water samples of different water sources along Santiago Island.

## Materials and methods

### Sampling and FLA culture

**Fig. 1:**
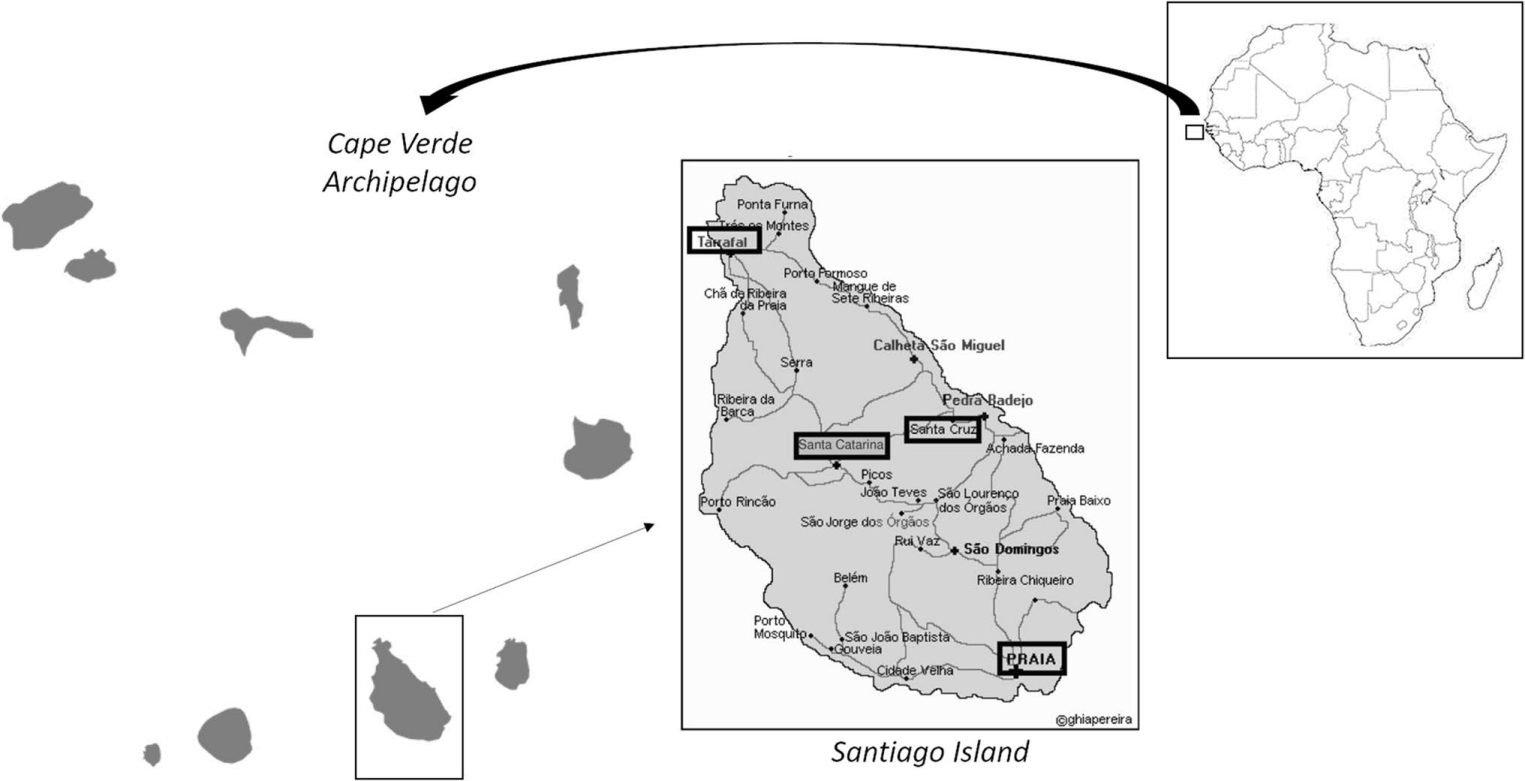
Cape Verde archipelago geographical situation and Santiago Island relative location ( adapted from Sousa-Ramos et al. 2021)

In order to reveal the presence of potentially pathogenic FLA in water samples of Santiago Island in Cape Verde (15°04′40″N 23°37′29″O), a total of 31 samples were collected from different towns across the island (Fig. 1). The evaluated samples correspond to tap (3/31), depurated (3/31), lake (2/31), swimming pool (4/31), conditioned air (1/31), sea (6/31) and irrigation waters (12/31), and they were collected in sterile bottles and maintained at 4 °C until further processing in the laboratory. As the samples were collected in Cape Verde and processed in Tenerife, Canary Islands (Spain), the processing time oscillated between 48 and 72 h after the sample collection. The samples collected from lakes, swimming pools, sea and depurated or irrigation systems were taken from the surface of each water body. The processing protocol consisted in filtering the water samples using a vacuum multiple system and 0.45 μm nitrocellulose filters (Pall, Madrid, Spain), and the filters were cultured inverted onto 2% non-nutrient agar (NNA) plates also seeded with heat-killed *Escherichia coli*. These plates were incubated at room temperature (~ 26 °C) and monitored daily. Those plates suspicious for FLA growth following morphological features (Page 1969) were cloned by dilution in new NNA plates until a monoxenic culture was obtained (Lorenzo-Morales et al. 2005; Reyes-Batlle et al. 2015).

### DNA extraction

For molecular characterization, DNA from positive samples was extracted from 1 to 2 ml of amoebic culture suspension. To obtain this amoeba suspension, 4 ml of Page’s Amoeba Solution (PAS) was added to the plate with the monoxenic amoeba culture. The plate was scraped and this suspension was centrifuged and the concentrated amoeba culture was directly placed into the Maxwell® 16 tissue DNA purification kit sample cartridge (Promega, Madrid, Spain) following the manufacturer’s instructions and as it has been previously described (Reyes-Batlle et al. 2019). The protozoa genomic DNA yield and purity were determined using the DS-11 Spectro-hotometer (DeNovix®, USA).

### PCR and molecular characterization of the obtained isolates

The amplification by PCR of the 18S rRNA gene from the obtained DNA was carried out using two universal primers for FLAf/r (Tsvetkova et al., 2004) and Ame f977/r1534 (Liang et al. 2010). On the other hand, to amplify the 18S rRNA DF3 fragment to distinguish *Acanthamoeba* genotypes, we have used the JDP-1f/JDP-2r (Schroeder et al. 2001).

Amplification reactions were performed with a total of 50 μl of mixture, containing 80 ng DNA, and the PCRs were carried out in 40 cycles with denaturation (95 °C, 30 s), annealing (FLAf/r 55 °C and Ame f977/r1534 62 °C, 30 s) and primer extension (72 °C, 30 s) for FLA universal primers (FLA and Ame). Nevertheless, for *Acanthamoeba* spp. primers, the 50 μl PCR mixture contains 40 ng of DNA yield and 35 cycles with denaturation (95 °C, 30 s), annealing (50 °C, 30 s) and primer extension (72 °C, 30 s). A primer extension of 7 min at 72 °C was maintained after the last cycle. PCRs products were analysed by electrophoresis through a 2% agarose gel and positive PCR products were sequenced by Macrogen Spain service (C/Martínez Villergas S2, Planta Baja, Of. 1, izda., Madrid, Spain). Different species were identified based on sequence homology analysis by comparison to the DNA sequences present in the Genbank database.

### Phylogenetic analyses

To proceed to sequence alignment, we have used the Mega X software program (Kumar et al. 2018; Tamura et al. 2004). The evolutionary history was inferred using the maximum likelihood method based on the Tamura-Nei model (Tamura and Nei 1993). The current analysis has involved 21 nucleotide sequences: 13 sample sequences and 8 GenBank standard sequences. The total of the ambiguous positions has been removed for each sequence pair.

## Results

From the total of the 31 analysed waters, 12 of them were positive for FLA growth (38.7%). However, we were able to genotype 13 FLA strains, being *Acanthamoeba* the most common identified genus (9/13; 69.2%), followed by *Vannella* spp. (2/13; 15.4%) and *Vermamoeba vermiformis* and *Stenamoeba dejonckheerei* (1/13; 7.7%).

The obtained sequences in the present study have been deposited in the GenBank database under the following accession numbers: MW757016- MW757028. All of them present ˃95% of homology with the available DNA sequences in this database (Table 1).

**Table 1:** Report of the FLA species isolated from the evaluated water sources of Cape Verde (*NNA* FLA growth in non-nutrient agar culture; *PCR* FLA detection by PCR; *homology (%) related to NCBI Database sequence)

Sample code	Locality	Water type	NNA	PCR	Genus/species	Homology (%)*
CVDW1	Santa Cruz	Reused wastewater	+	+	*Vannella croatica*	≥ 95%
CVLW1	Varzea, Praia	Recreational fountain	+	+	*Acanthamoeba* sp. T4	≥ 95%
CVSP2	Cidade Velha, Ribeira Grande De Santiago	Swimming pool	+	+	*Vannella* sp*.*	≥ 95%
CVSW1	Tarrafal	Sea	+	+	*Acanthamoeba lenticulata* T5	≥ 95%
CVSW4	Praia	Sea	+	+	*Acanthamoeba* sp. T4	≥ 95%
CVSW5	Praia	Sea	+	+	*Stenamoeba dejonckheerei*	≥ 95%
CVIW1	Cidade Velha, Ribeira Grande De Santiago	Irrigation	+	+	*Acanthamoeba* sp. T4	≥ 95%
CVIW2	Cidade Velha, Ribeira Grande De Santiago	Irrigation	+	+	*Acanthamoeba* sp. T4	≥ 95%
CVIW3	Santa Cruz	Irrigation	+	+	*Acanthamoeba lenticulata* T5	≥ 95%
			+	+	*Vermamoeba vermiformis*	≥ 95%
CVIW4	Santa Cruz	Irrigation	+	+	*Acanthamoeba* sp. T4	≥ 95%
CVIW9	Santa Cruz	Irrigation	+	+	*Acanthamoeba* sp. T4	≥ 95%
CVIW11	Santa Cruz	Irrigation	+	+	*Acanthamoeba culbertsoni* T4	≥ 95%

**Fig. 2:**
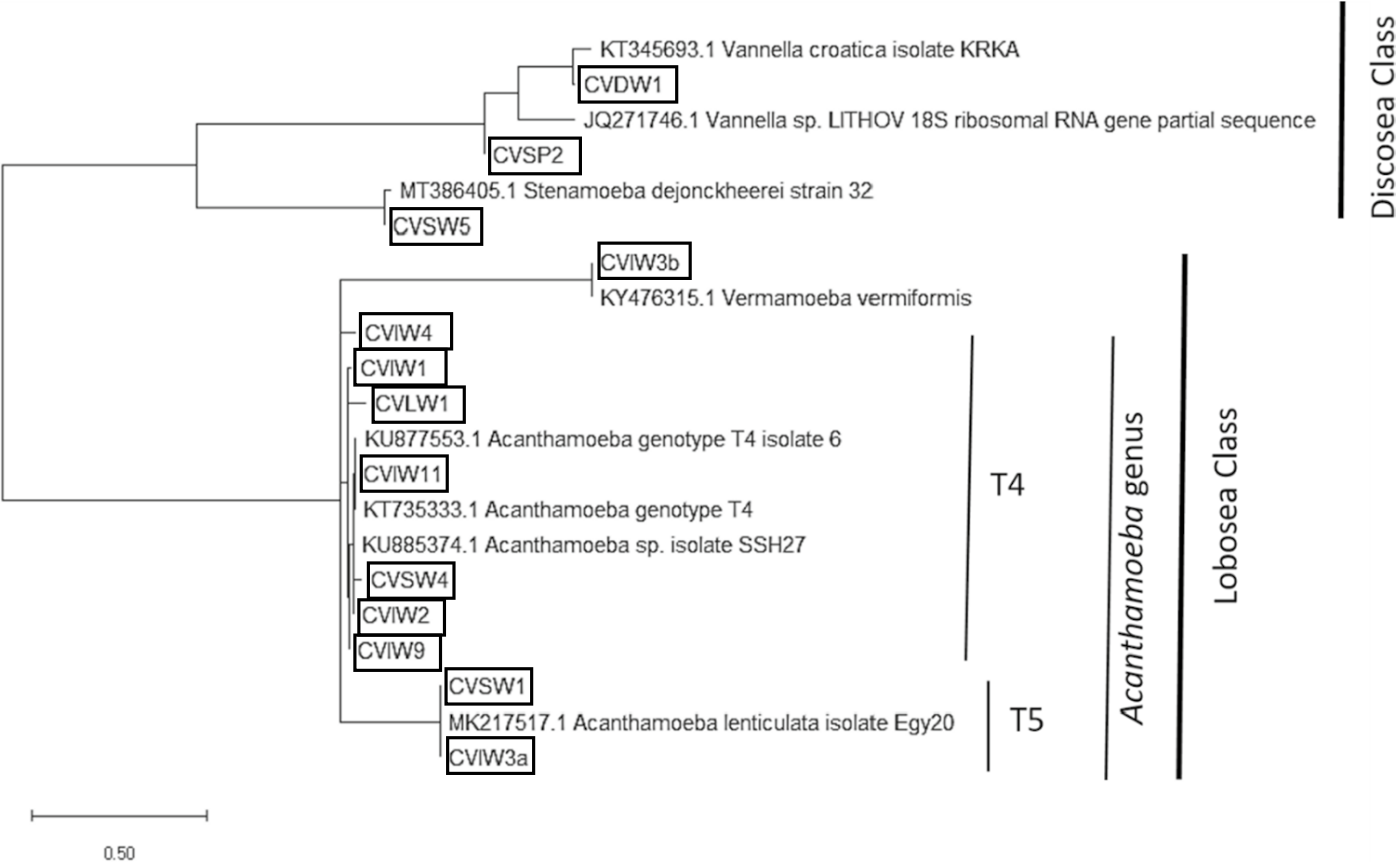
Phylogenetic relationship of the FLA strains isolated in the present study. The isolates obtained in the present study are identified in boxes

The phylogenetic relationship of the FLA strains isolated in the present study is represented in Fig. 2, where the Discosea species (*Stenamoeba* spp. and *Vannella* spp.) and the Lobosea species (*Acanthamoeba* spp. and *Vermamoeba* spp.) are well differentiated. The evolutionary history was inferred by using the maximum likelihood method based on the Tamura-Nei model (Tamura and Nei 1993). The initial tree for the heuristic search was obtained automatically by applying Neighbor-Join and BioNJ algorithms to a matrix of pairwise distances estimated using the maximum composite likelihood (MCL) approach, and then selecting the topology with a superior log-likelihood value. The tree is drawn to scale, with branch lengths measured in the number of substitutions per site. There were a total of 2328 positions in the final dataset and the evolutionary analyses were conducted in MEGA X (Kumar et al. 2018).

## Discussion

Cape Verde has high levels of poverty and unemployment, partly attributable to a lack of obvious economic growth opportunities and a scarcity of resources, particularly water (Paul D. Coverdell 2009). The largest island, both in size and population, is Santiago, where Praia, the capital, is located. These islands with a volcanic origin count with some of the windiest beaches in the world, and they vary widely in terrain (Paul D. Coverdell 2009). Water shortages and successive droughts have greatly weakened crop production capacity over the last century (Paul D. Coverdell 2009). Santiago has vegetation-clad (cloud forests) where the dense moisture condenses and soaks the plants and soil and favours agriculture (Paul D. Coverdell 2009). Therefore, taking into account the importance of having appropriate quality water sources, it is important to go deep into the possible microbial contamination, such as bacteria, viruses or protozoa.

FLA have been considered amphizoic protozoa, which means that these protozoa do not require a host organism to be able to survive (Cateau et al. 2014). Consequently, they are considered an emerging group of opportunistic pathogens (Lorenzo-Morales et al. 2015) since they represent a health risk not only because they are capable of causing several diseases in humans and other animals, but also because they act as vehicles for potentially pathogenic bacteria (Siddiqui and Khan 2012). In the present work, we have isolated *Acanthamoeba* spp. (69.2%); *Vannella* spp. (15.4%); *Vermamoeba vermiformis* (7.7%) and *Stenamoeba dejonckheerei* (7.7%) in different water sources of Santiago Island. Up to now, in Europe and Africa, there is no legislation related to the presence of FLA in water bodies. However, Australia possesses a Drinking Water Guideline where they gather the National Water Quality Management Strategies (Australian drinking water guidelines 6,2011 national water quality management strategy). In this document, even though there is no guideline value for *Acanthamoeba*, *Vannella, V. vermiformis* and *Stenamoeba* species in drinking water, they named *Acanthamoeba* and *Vannella* in a different manner.

Most of the *Acanthamoeba* species are capable to produce cerebral infections known as granulomatous amoebic encephalitis (GAE), corneal infection, *Acanthamoeba* keratitis (AK) or both (Lorenzo-Morales et al. 2015). In the referred guideline, they remark that the relative importance of water as a source of *Acanthamoeba* infection is unknown. Moreover, they highlight the wide distribution of *Acanthamoeba* in a natural environment such as soil, airborne dust and water and how the delays in the diagnosis of GAE and AK cases have made it difficult to investigate possible sources of infection, while the lack of a stable classification of *Acanthamoeba* makes difficult the identification of individual isolates, including the matching of amoebae from infections with organisms from the environment (Australian drinking water guidelines 6,2011 national water quality management strategy). Even though the regular monitoring for *Acanthamoeba* is not appropriate in this guideline, they enhance that these organisms need to be considered when planning the maintenance of eyewash stations that use main water. On the other hand, *Vannella* spp. is only contemplated as a microorganism which could cause taste and odour problems. So far, there is no evidence related to the pathogenicity of *Vannella* spp. However, it is well known that this genus can facilitate the growth of bacteria (Loret et al. 2008; Schulz et al. 2015) such as *Legionella* (Kuroki et al. 1998) or other human pathogenic organisms (Scheid 2007).

In contrast, *V. vermiformis* has been recently reported as one of the most prevalent and thermotolerant FLA (Reyes-Batlle et al. 2016; Siddiqui and Khan 2021). *V. vermiformis* pathogenicity is not only due to its capability to produce corneal, encephalitic or epithelial infections, but also to its numerous reported relationships with pathogenic bacteria (Scheid 2019; Sousa-Ramos et al. 2021). On the other hand, until now, no pathogenicity has been associated to *Stenamoeba* genus, which was finally defined in 2007 as a member of Thecamoebida (Discosea) (Adl et al. 2019). However, this new FLA species was isolated from fresh-water reservoirs at 37 °C, a temperature which could indicate a potentially pathogenic risk. This is the third report of the new species *Stenamoeba dejonckheerei* worldwide (Borquez-Román et al. 2020; Lares-Jiménez et al. 2018; Sousa-Ramos et al. 2021) and the second time in Cape Verde Archipelago (Sousa-Ramos et al. 2021).

## Conclusions

In the present work, according to a previous study which analysed soil samples from Santiago Island (Sousa-Ramos et al. 2021), we have reported the presence of FLA in environmental sources, specifically in water samples. The presence of *Acanthamoeba* species, most of them belonging to the virulent T4 genotype (7 isolates), and *V. vermiformis*, enhances the importance to control the protozoa contamination in human-related water sources. In fact, we should take care about not only this pathogenic potential, but also the capability of these protozoa species to transport other human pathogens, also shared by *Vannella* spp. Finally, as *S. dejonckheerei* is a recently discovered species, we consider it important to notify its presence in human-related environments.
